# Immunohistochemical investigation of Foxp3 expression in the intestine in healthy and diseased dogs

**DOI:** 10.1186/1297-9716-43-23

**Published:** 2012-03-22

**Authors:** Johannes Junginger, Ulrike Schwittlick, Frederik Lemensieck, Ingo Nolte, Marion Hewicker-Trautwein

**Affiliations:** 1 Institute of Pathology, University of Veterinary Medicine Hannover, Bünteweg 17, D-30559 Hannover, Germany; 2 Small Animal Clinic Duisburg-Asterlagen, Dr.-Detlev-Karsten-Rohwedder-Str. 11, D-47228 Duisburg-Rheinhausen, Germany; 3 Small Animal Clinic, University of Veterinary Medicine Hannover, Bünteweg 9, D-30559 Hannover, Germany

## Abstract

Intestinal immune regulation including development of oral tolerance is of great importance for the maintenance of intestinal homeostasis. Concerning this, regulatory T cells (Tregs) occupy a pivotal role in cell-mediated immunosuppression. Dysregulation of mucosal immunology leading to an abnormal interaction with commensal bacteria is suggested to play a key role in the pathogenesis of Inflammatory Bowel Disease (IBD) in men and dogs. The aim of this study was to characterise the expression of Foxp3 in the normal canine gut of 18 dogs (mean age: 6.03 years), in 16 dogs suffering from IBD (mean age: 5.05 years), and of 6 dogs with intestinal nematode infection (mean age: 0.87 years) using immunohistochemistry. In the duodenum, Tregs in healthy dogs declined from villi (median: 10.67/62 500 μm^2^) to crypts (median: 1.89/62 500 μm^2^). Tregs were further increased in the villi of middle-aged dogs (median: 18.92/62 500 μm^2^) in contrast to juvenile (median: 3.50/62 500 μm^2^) and old (median: 9.56/62 500 μm^2^) individuals. Compared to healthy controls, animals suffering from IBD revealed reduced numbers of Tregs in duodenal villi (median: 4.13/62 500 μm^2^). Dogs with intestinal nematode infection displayed increased numbers of Tregs (median: 21.06/62 500 μm^2^) compared to healthy animals.

Age-related changes indicate a progressive establishment of oral tolerance and immunosenescence in the canine elderly. The results further suggest that a defect in Treg homeostasis may be involved in the pathogenesis of canine IBD. In contrast, increased numbers of Tregs in the duodenum may be due to nematode infection.

## Introduction

The vertebrate's gut is perpetually exposed to an enormous amount of different microorganisms and food proteins with antigenic properties that are essential for the individual's life. Concerning this high antigenic load, a complex immunological network has been developed representing an effective protection composed of more structured areas (lymphoid aggregates, Peyer's patches, lymphoglandular complexes, and mesenteric lymph nodes) as well as diffuse parts (lamina propria leucocytes and intraepithelial lymphocytes) [1-4]. Due to the intestinal colocalisation of commensal bacteria and pathogens, mechanisms preventing specific immune responses against orally administrated antigens (e.g. food proteins and commensal bacteria) termed as oral tolerance are as much as essential as the induction of specific immune responses against pathogenic organisms.

In general, immune regulation can be either mediated by regulatory cytokines (e.g. tissue growth factor (TGF)-β, interleukin (IL)-10) or cellular interactions. Today, it is well-known that specialised T helper cells with regulatory properties (Tregs) play an important part in immune regulation [5,6]. Depletion of these cells or mutations of related transcription factors provokes severe cases of autoimmune diseases and chronic inflammatory disorders as described in rats, mice, and men [7-10]. Furthermore, oral tolerance is supposed to be mainly mediated by the induction and expansion of Tregs in the context of specialised tolerogenic dendritic cells in the gut [11-13]. Alongside their regulatory capacity, Tregs are characterised by the expression of CD4, CD25 [7], and the highly conserved transcription factor Forkhead box P3 (Foxp3) [14] serving a pivotal role in stabilising their regulatory properties [15].

Canine idiopathic Inflammatory Bowel Disease (IBD) is a term used for a variety of disorders characterised by chronic persistent or recurrent gastrointestinal signs (e.g. vomiting, diarrhoea, and weight loss) with no underlying cause (e.g. parasitosis, bacterial infection, food allergy, lymphoma, or lymphangiectasia) and histological evidence of mucosal inflammatory infiltration [16-18].

In humans, idiopathic IBD is known as Crohn's disease (CD) or ulcerative colitis (UC) [19,20]. CD is characterised by transmural neutrophilic inflammation followed by granuloma formation commonly present in the terminal jejunum and ileum and is often complicated by intestinal or anal fistulae, strictures, obstructions, or intestinal perforations [21]. In UC, neutrophilic infiltration is restricted to the colonic and rectal mucosa and is associated with ulceration and crypt abscesses. In addition to intestinal lesions, extraintestinal manifestations (e.g. arthritis, renal disease, pyrexia, mucocutaneous lesions, hepatobiliary complications, or osteopenia) are often reported in human IBD [22].

In dogs, mucosal inflammation is the main histopathological feature of idiopathic IBD and variants are classically distinguished regarding their dominant cellular infiltrates [17,23]. Lymphoplasmacytic enteritis (LPE) represents the most common form of idiopathic IBD in dogs [24]. Additionally, eosinophilic gastroenteritis (EGE) characterised by a mixed infiltration of inflammatory cells with dominance of eosinophilic granulocytes is the second most frequently diagnosed form of canine IBD. Furthermore, several variants of LPE and EGE exist since inflammation can be restricted to one or more anatomical sites (e.g. stomach, small intestine, or colon). Additionally, histiocytic ulcerative colitis (HUC) is a rare colonic disorder reported in boxers that is characterised by the histological presence of PAS-positive macrophages within the colonic mucosa [25,26] that is supposed to be associated with *Escherichia coli *[27]. Furthermore, idiopathic granulomatous intestinal inflammation characterised by infiltration of macrophages with granuloma formation similar to CD is rarely reported in dogs [28,29]. In contrast to human IBD, complications are rare in canine patients and only thrombocytopenia is discussed as an extraintestinal manifestation of IBD in dogs [30]. Although canine idiopathic IBD differs from CD and UC in humans in several aspects, a common pathogenesis is suggested. Idiopathic IBD is supposed to develop in a multilayer model including genetic factors [31-35], dysregulation of the immune system, and disturbances of the intestinal microflora leading to a breakdown of immunological tolerance to luminal antigens [36,37].

Parasitic infections constitute common intestinal diseases with zoonotic potential in companion animals [38,39]. Possible clinical signs in dogs include vomiting, diarrhoea, abdominal pain, and weight loss, but clinical symptoms may be absent. Histopathologically, affected tissues reveal varying degrees of cellular infiltrations with prominence of eosinophilic granulocytes [40] and therefore share some histological features with canine idiopathic EGE. Immunologically, intestinal helminths trigger downregulation of the immune system ensuring longer survival in the host [41,42]. In murine models of nematode infection, Foxp3+ Tregs expand after application of parasites and correlate with the period of worm survival, but decrease after application of anti-CD25 antibodies that is associated with increased intestinal pathology [43-45]. Furthermore, excretory-secretory products of several parasites (*Heligmosomoides polygyrus, Haemonchus contortus*, and *Teladorsagia circumcincta*) are known to drive TGF-β-like signalling resulting in expansion of murine Foxp3+ Tregs in vitro and in the gut-associated lymphoid tissue (GALT) in vivo [46]. Interestingly, the administration of parasitic antigens is discussed as a possible treatment of human IBD due to their regulatory properties [47,48].

Since the availability of specific antibodies, Foxp3 has been established as a specific marker for Tregs in different species [49,50] and the literature concerning regulatory T cells in domestic animals has been increased [51]. In cats, CD4+ CD25+ Tregs have been characterised in detail in the context of the pathogenesis of feline immunodeficiency virus [52-55]. Tregs are also described and characterised in pigs [56-58], especially in linkage to foetal tolerance [59], renal and cardiac allotransplantation [60-62], and infection with porcine reproductive and respiratory syndrome virus [63,64]. Expression of Foxp3 is also reported in cows. Most of bovine Foxp3-positive cells coexpress CD4 and CD25 [65], although minor populations of CD8β+ and γδ+ Foxp3+ T cells with Foxp3 expression have been described [66]. However, the regulatory properties of CD4+ CD25+ Foxp3+ bovine T cells are controversially discussed, since γδ+ T cells and CD14+ monocytes but not CD4+ CD25+ Foxp3+ cells exhibited regulatory function in one study [67]. Additionally, ovine Foxp3 has recently been described and Foxp3 expressing cells are significantly increased in the skin of sheep infected with *Psoroptes ovis *[68]. Other species in which Foxp3 expression is described include the horse [69], baboon [70,71], macaque [72], chimpanzee [73], harbour seals and walrus [74], and zebrafish [75].

In dogs, the principle of peripheral tolerance was first suggested in 1976 as a specific population of T cells seeming to prevent graft-versus-host reaction [76]. In 2007, a subset of CD4+ T cells in blood and/or lymph nodes of dogs was detected using anti-mouse/rat Foxp3 monoclonal antibodies (clone FJK-16s) and authors suggested crossreactivity of these antibodies with canine Foxp3 [49]. Since then, the crossreactivity of this anti-mouse/rat Foxp3 antibody with canine Foxp3 has been confirmed by investigation of cell lines that overexpress the canine Foxp3 gene [77]. Additionally to Foxp3, specific antibodies against canine CD25 have been available for a few years [77-79] and therefore CD4+ CD25+ Foxp3+ T cells have been described in the canine peripheral blood [77,80]. Additionally, canine Foxp3+ Tregs were investigated in the context of neoplasia, atopic dermatitis, and adverse food reactions [49,81-87]. Recently, the regulatory function of canine CD4+ CD25+ Foxp3+ T cells was described in vitro [79,80]. Furthermore, there is evidence for heterogeneity of canine Tregs since those with high expression of Foxp3 are possibly activated Tregs in contrast to Tregs with intermediate expression of Foxp3, which are suggested to be a more heterogeneous population of predominantly activated conventional T cells [80]. Regarding the canine gastrointestinal tract, there is only one report about the expression of cytokines and transcription factors of different T cell subsets including Tregs in the duodenum of dogs. In this study, no alterations in gene expression were present in dogs with cutaneous food hypersensitivity compared to healthy controls [86]. Until today, no investigations of Foxp3 expression in canine idiopathic IBD, nor in canine nematode infections have been available.

The aim of the study was to investigate the expression of Foxp3 in the canine GALT in healthy animals and in dogs with chronic idiopathic IBD. Additionally, we investigated cases of canine intestinal nematode infections that share some histopathological features with canine idiopathic EGE.

## Materials and methods

### Animals

For the evaluation of Foxp3-positive cells in the normal canine gut, 18 dogs without gastrointestinal diseases routinely submitted for necropsy to the Institute of Pathology (University of Veterinary Medicine, Hannover) were used (Table 1). These dogs were presented to the Small Animal Clinic of the University of Veterinary Medicine (Hannover) because of other problems than gastrointestinal symptoms. A detailed clinical workup (e.g. case history, clinical investigation, haematology, serum biochemistry, ultrasonics, and taking X-rays and MRI if necessary) was performed by the clinicians on these dogs and no gastrointestinal symptoms were present during this procedure. Additionally, only dogs without any pathomorphological signs of gastrointestinal disorders during necropsy were used as healthy controls. Tissue samples collected from different locations (gastric fundus, descending duodenum, middle of jejunum, ileum, descending colon, and mesenteric lymph node) were taken as soon as possible after euthanasia (approximately between 15 and 30 minutes post mortem), being immediately fixed in 10% neutral buffered formalin, and embedded in paraffin. Animals were grouped as juvenile (0.08 to 0.99 years), middle-aged (1.94 to 6.25 years), and old individuals (8.98 to 15.22 years).

**Table 1:** Dogs without gastrointestinal diseases.

**No**.	Breed	Age (years)	Age group	WSAVA total	WSAVA part	Reason for euthanasia
1	Leonberger	0.08	juvenile	1	1	Oesophageal dilatation
2	Labrador	0.20	juvenile	4	2	Oesophageal dilatation
3	Alsatian	0.22	juvenile	0	0	Seizures
4	Pinscher	0.52	juvenile	3	1	Ataxia
5	Briard	0.99	juvenile	0	0	Ataxia
6	Jack Russel Terrier	1.94	middle-aged	3	3	Right hemiparesis
7	Siberian Husky	4.00	middle-aged	6	3	Seizures
8	Kuvasz	4.28	middle-aged	0	0	Vertebral sarcoma
9	Australian Shepherd	4.43	middle-aged	3	3	GME
10	Pointer	5.85	middle-aged	3	0	Hypophyseal adenoma
11	Pyrenean Shepherd	6.09	middle-aged	2	2	Spinal disk herniation
12	Yorkshire Terrier	6.25	middle-aged	4	4	NLE
13	Beagle	8.98	old	4	4	Tonsillar SCC
14	Crossbreed	10.16	old	2	2	GME
15	Crossbreed	12.00	old	4	3	Haemangiosarcoma
16	Alsatian	12.85	old	6	4	Spinal disk herniation
17	Crossbreed	14.77	old	3	2	HCM
18	Crossbreed	15.22	old	1	1	Pancreatic adenocarcinoma

WSAVA = histopathological score regarding the guidelines for evaluation and scoring of canine intestinal inflammation of the World Small Animal Veterinary Association (WSAVA) Gastrointestinal Standardization Group. WSAVA total = WSAVA score of the total gastrointestinal tract (including stomach, duodenum, jejunum, ileum, and colon). WSAVA part = WSAVA score of the stomach, duodenum, and colon (for comparison with WSAVA scores of dogs with gastrointestinal diseases). *GME *granulomatous meningoencephalitis. *NLE *necrotizing leucoencephalitis. *SCC *squamous cell carcinoma. *HCM *hypertrophic cardiomyopathy.

For the investigation of Tregs in canine IBD, gastrointestinal biopsy specimen were obtained from 16 dogs suffering from chronic gastrointestinal disorders of unknown aetiology (ages ranging from 2.00 to 7.22 years) that were admitted to the Small Animal Clinic of the University of Veterinary Medicine (Hannover) as well as to another Small Animal Clinic (Duisburg Asterlagen, Table 2). Clinically, dogs were presented with chronic (at least 3 weeks) or recurrent vomiting (no. 1- 4, 6- 8, 13, 15, and 16) and/or diarrhoea (no. 1, 2, 4-12, 14, and 15) partially in conjunction with emaciation (no. 7, 9, 12, 14, and 16). Detailed clinical workup was performed for every patient (including clinical examination, taking X-rays, ultrasonics, haematology, serum biochemistry comprising determination of cobalamin, urinanalysis, microbiology and parasitology of faecal samples, and antimicrobial therapy including application of metronidazole). Several endoscopical biopsies (at least four per localisation) taken from the gastric fundus (*n *= 16), duodenum (*n *= 16), and colon (*n *= 8; no. 1, 2, 4, 9, 12, 13, 15, and 16) during routine diagnostics were fixed in 10% neutral buffered formalin and embedded in paraffin.

**Table 2:** Dogs with inflammatory gastrointestinal diseases.

**No**.	Breed	Age (years)	Age group	WSAVA score	Disease
1	Crossbreed	2.00	middle-aged	6	EGE
2	Hungarian Vizslar	2.75	middle-aged	14.5	EGEC
3	Crossbreed	3.00	middle-aged	7	EE
4	Crossbreed	4.60	middle-aged	12	EGEC
5	Border Collie	4.76	middle-aged	5	EE
6	Irish Terrier	7.00	middle-aged	7	EGE
7	Crossbreed	7.00	middle-aged	5.5	EGE
8	Hovawart	2.33	middle-aged	8	LPGEC
9	Malinois	4.00	middle-aged	8.5	LPGE
10	Belgian Shepherd	4.50	middle-aged	7.5	LPGE
11	Jack Russel Terrier	5.24	middle-aged	8	LPGEC
12	Boxer	6.00	middle-aged	9	LPEC
13	Crossbreed	6.50	middle-aged	7.5	LPGE
14	WHWT	6.82	middle-aged	15.5	LPGEC
15	Poodle	7.00	middle-aged	4.5	LPGEC
16	Crossbreed	7.22	middle-aged	4.5	LPGE
17	Fox Terrier	0.18	juvenile	11	NI
18	Fox Terrier	0.18	juvenile	8	NI
19	Beagle	1.17	juvenile	3	NI
20	Beagle	1.17	juvenile	6	NI
21	Beagle	1.25	juvenile	10	NI
22	Beagle	1.25	juvenile	11	NI

WHWT = West Highland White Terrier. EE = IBD, eosinophilic enteritis. EGE = IBD, eosinophilic gastroenteritis. EGEC = IBD, eosinophilic gastroenterocolitis. LPE = IBD, lymphoplasmacytic enteritis. LPGE = IBD, lymphoplasmacytic gastroenteritis. LPEC = IBD, lymphoplasmacytic enterocolitis. LPGEC = IBD, lymphoplasmacytic gastroenterocolitis. NI = intestinal nematode infection.

Furthermore, 6 dogs with spontaneous intestinal nematode infection detected by macroscopical and/or microscopical findings (age ranges from 0.18 to 1.25 years) were evaluated (Table 2). Clinically, no gastrointestinal symptoms were present in these dogs. Fox Terrier dogs (no. 17 and 18) were euthanised due to progressive paraparesis and routinely submitted for necropsy to the Institute of Pathology (University of Veterinary Medicine, Hannover). Beagle dogs (no. 19-22) were humanely destroyed for reasons unrelated to the present study followed by macroscopical and histopathological examination. Tissue specimens were taken as soon as possible after euthanasia (approximately between 15 and 30 min post mortem) from gastric fundus, descending duodenum, and descending colon and each were immediately processed as described above.

### Histopathological examination

**Figure 1:**
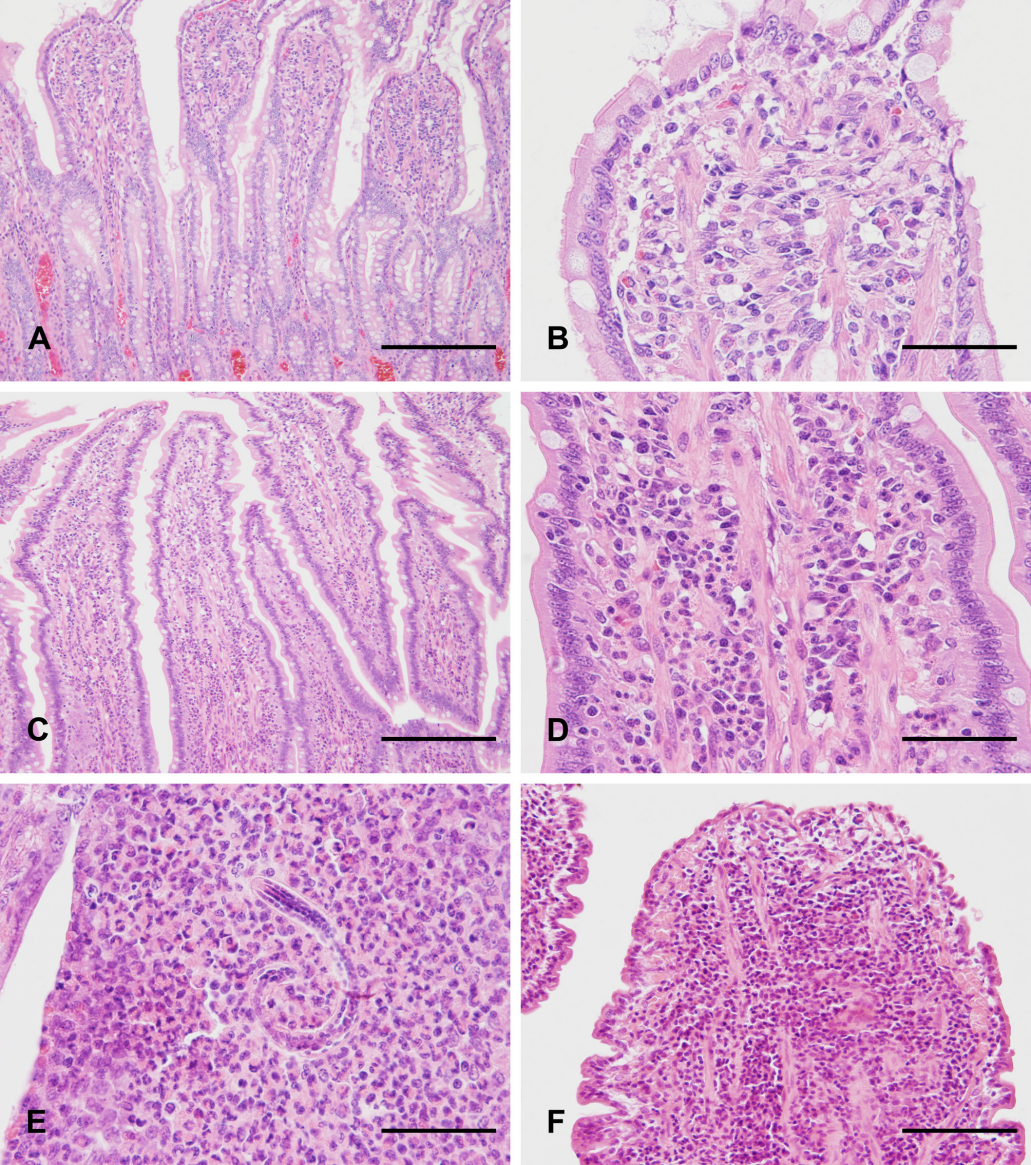
**Histology of dogs without gastrointestinal symptoms and of dogs suffering from gastrointestinal diseases**. Intestinal samples of dogs used as healthy controls (A: dog no. 8, bar: 200 μm; B: dog no. 8, bar: 50 μm) and of dogs with intestinal nematode infection (C: dog no. 20, bar: 200 μm; D: dog no. 20, bar: 50 μm) revealed comparable freshness to intestinal biopsy specimens. In dogs with intestinal nematode infection, mucosal inflammation was dominated by eosinophilic granulocytes in addition to increased amounts of lymphocytes, plasma cells, and neutrophils (D: dog no. 20, bar: 50 μm). Furthermore, parasitic structures resembling nematode larvae were occasionally present within inflammatory foci (E: dog no. 20, bar: 50 μm). In dogs with LPE, intestinal mucosa was infiltrated by lymphocytes and plasma cells (F: dog no. 9, bar: 100 μm).

For histopathological examination, 2-4 μm thin sections were prepared and stained with haematoxylin and eosin (H&E) by standard histological procedures. Slices were independently evaluated by 3 pathologists (JJ, US, MHT) using light microscopes (Carl Zeiss) according to the histopathological guidelines for the evaluation and scoring of canine intestinal inflammation recently published by the World Small Animal Veterinary Association (WSAVA) Gastrointestinal Standardisation Group [88]. Concerning dogs used as healthy controls and dogs with intestinal nematode infection, only samples with no or minimal signs of autolysis were included in this study ensuring that they were comparable to endoscopical biopsy specimens (Figure 1a-d).

### Immunohistochemistry

For immunohistochemistry, 2-4 μm thin sections were dewaxed in Rotihistol (Roth C. GmbH & Co. KG, Karlsruhe, Germany) and rehydrated in descending ethanol series. Blocking of endogenous peroxidase was achieved by incubation of the sections in H_2_O_2 _0.5% in ethanol for 30 min. Antigen retrieval was performed with citrate buffer (pH 6.0, 20 min, 95°C) followed by the application of normal rabbit serum for 30 min. Sections were then incubated with anti-mouse/rat Foxp3 antibodies (1 in 400 dilution; clone FJK-16s, eBioscience, San Diego, CA) overnight at 4°C that have shown to detect canine Foxp3 using flow cytometry [49,77] and immunohistochemistry [89], followed by secondary antibody application (biotinylated rabbit anti-rat IgG [H + L]; Vector Laboratories, Burlingame, California) for 30 min. Subsequently, avidin-biotin complex (Vector Laboratories) was utilised for 30 min. Accordingly, biotinylated tyramine was applied to intensify the reaction followed by a second incubation with an avidin-biotin complex. Between each step, sections were rinsed three times in phosphate buffered saline (PBS; pH 7.2, 0.15 M). 3,3'-diaminobenzidine and H_2_O_2 _was applied for 5 min to generate a brown colour reaction. Sections were counterstained with Mayer haematoxylin. Sections of normal canine thymus and mandibular lymph node were used as positive controls. The primary antibody solution was substituted with normal rat serum as a negative control.

### Examination of sections

Cells revealing clear lymphocyte morphology with distinct nuclear staining for Foxp3 but unstained cytoplasm (interpreted as Tregs) were quantified in different compartments (gastric fundus: lamina propria; small intestine: villus, basal crypt area, villus-crypt junction; large intestine: apical crypt area, basal crypt area) using a light microscope (Carl Zeiss), a × 40 objective, a × 10 eyepiece, and a square eyepiece graticule (10 × 10 squares, with a total area of 62 500 μm^2^). Ten appropriate sites were chosen for each compartment and arithmetic means were calculated for each one. The results are expressed as positive cells per 62 500 μm^2^.

### Statistical analysis

Statistical analyses were performed using R version 2.13.1 (The R Foundation for Statistical Computing, Vienna, Austria). Primarily, each group was assessed for normal distribution using a histogram, Q-Q plot, and Shapiro-Wilk test. Kruskal-Wallis test and/or Wilcoxon rank-sum test were used to evaluate differences in WSAVA scores between different age groups in healthy dogs as well as between dogs with gastrointestinal diseases and controls. Furthermore, statistical significant differences in the numbers of Foxp3-positive cells between groups concerning different ages, localisations, and compartments as well as between diseased dogs and healthy controls were assessed using the Kruskal-Wallis test and Wilcoxon rank-sum test for independent samples or the Friedman test and Wilcoxon signed-rank test for related ones. Furthermore, the Spearman's rank correlation coefficient was used to investigate the relationship between intestinal Foxp3-positive cells in dogs with IBD and the WSAVA score for the appropriate intestinal section. *P *values ≤ 0.05 were defined as statistically significant.

## Results

### Histopathological findings

In dogs lacking clinical and pathomorphological signs of gastrointestinal diseases, mild histopathological findings were present in the gut (e.g. mild increases in lamina propria lymphocytes and plasma cells, crypt abscesses, crypt dilation, and mucosal fibrosis). These findings are summarised in Table 1 given as WSAVA scores. In these animals, no significant differences between the WSAVA scores of different groups of age were present.

Endoscopical biopsy specimens of dogs with chronic or recurrent gastrointestinal disorders revealed histopathological evidence of mucosal inflammation (Figure 1f). Therefore idiopathic IBD was diagnosed since other possible aetiologies were excluded (e.g. parasitic disease, bacterial infection, food allergy, antibiotic-responsive diarrhoea, lymphangiectasia, or neoplasia). Cellular infiltrates were either composed of lymphocytes and plasma cells (*n *= 9) or mixed inflammatory cells with prominence of eosinophilic granulocytes (*n *= 7).

In dogs with intestinal nematode infection, mucosal inflammation was dominated by eosinophilic granulocytes in addition to increased amounts of lymphocytes, plasma cells, and neutrophils (Figure 1d). Furthermore, parasitic structures resembling nematode larvae were occasionally present within inflammatory foci (Figure 1e).

Compared to healthy controls, WSAVA (summarised in Table 2) were significantly increased in dogs suffering from IBD (*p *< 0.001) and in animals with intestinal nematode infections (*p *< 0.01).

### Foxp3 expression in the canine gut of healthy dogs

**Figure 2:**
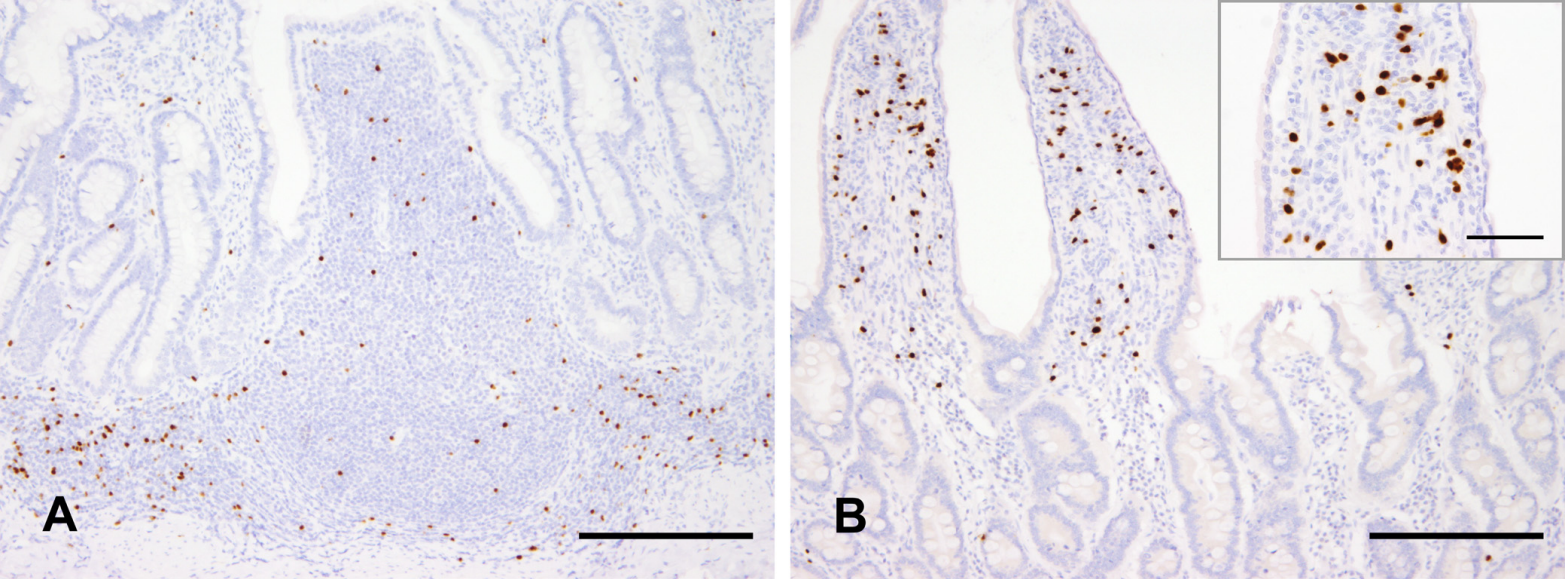
**Foxp3 expression in the canine healthy GALT**. In canine Peyer's patches, Foxp3-positive cells were numerously present in interfollicular areas and lower amounts were visible in dome areas. Only few cells were located at the margin of B cell follicles (A: jejunum, dog no. 10, bar: 200 μm). Regarding the small intestinal lamina propria, Foxp3-expressing lymphocytes were more numerous in the villus compared to crypts (B: ileum, dog no. 8, bar: 200 μm, inset bar: 50 μm).

In healthy dogs, Foxp3 was expressed in both structured and diffuse parts of the canine GALT. Concerning Peyer's patches, Foxp3-positive cells were numerously present in interfollicular areas and lower amounts were visible in dome areas (Figure 2a). Only a few cells were located at the margin of B cell follicles. In gastric lymphoid aggregates as well as in colonic lymphoglandular complexes, Foxp3-positive cells were randomly distributed around the follicles. Mesenteric lymph nodes revealed high amounts of Foxp3-positive Tregs that were largely present in the paracortex as well as in the medullary sinus. In diffuse parts of the canine GALT, Foxp3 was expressed by both lamina propria lymphocytes as well as intraepithelial lymphocytes.

**Figure 3:**
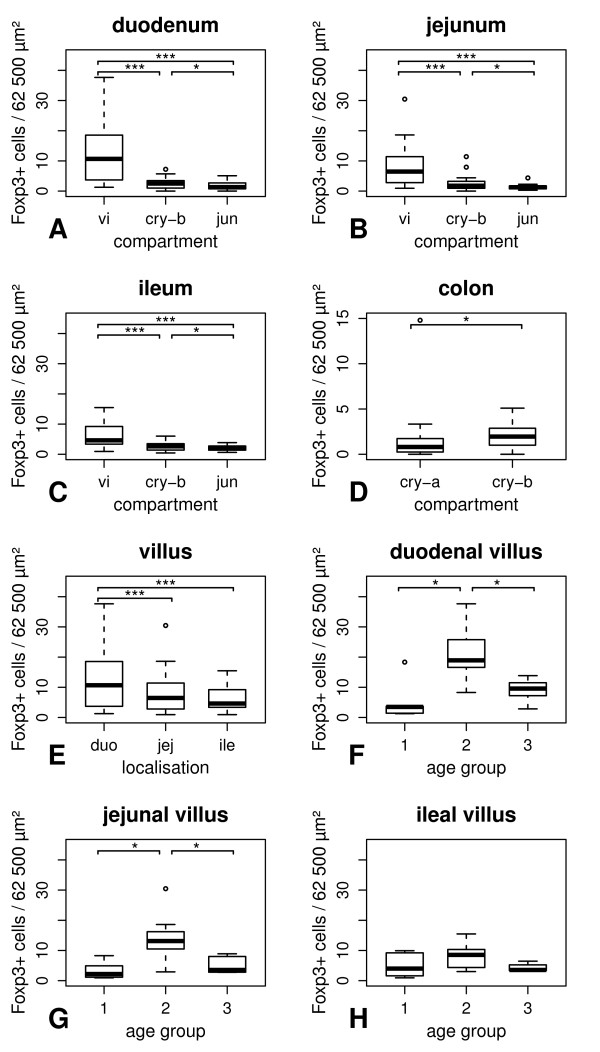
**Intestinal Foxp3 expression in dogs without gastrointestinal diseases**. Without consideration of their age, numbers of lamina propria Foxp3-positive lymphocytes were significantly increased in the villus compared to the basal crypt area as well as the villus-crypt junction and the density of Foxp3-positive lymphocytes was significantly higher in the basal crypt area than in the villus-crypt junction (A-C). In the colon, Foxp3-positive cells were increased in the basal crypt area compared to the apical part (D). Regarding the villus, lamina propria Foxp3-positive cells were significantly decreased in the jejunum and the ileum compared to the duodenum in healthy dogs disregarding their age (E). Concerning age-related changes, numbers of villus Foxp3-positive lymphocytes were significantly increased in middle-aged dogs compared to juvenile as well as old animals in the duodenum and the jejunum (F-G). In contrast, age-related changes were absent in the ileum (H). vi = villus. cry-b = basal crypt area. jun = villus-crypt junction. cry-a = apical crypt area. duo = duodenum. jej = jejunum. ile = Ileum. 1 = juvenile. 2 = middle-aged. 3 = old. * = *p *< 0.05. *** = *p *< 0.001.

Numbers of Foxp3 expressing lymphocytes in the gut of healthy dogs are summarised in Table 3 given as medians per group. Without consideration of different age groups, the numbers of lamina propria Foxp3-positive lymphocytes were significantly increased in villi compared to basal crypt areas and the villus-crypt junction (*p *< 0.001, Figure 2b and 3a-c) in healthy animals. Furthermore, the density of Foxp3-positive lymphocytes was significantly higher in the basal crypt area than in the villus-crypt junction (*p *< 0.05). In the colon, Foxp3-expressing cells were increased in the basal crypt areas compared to the apical areas (*p *< 0.05, Figure 3d).

**Table 3:** Foxp3 expressing lymphocytes in dogs without gastrointestinal diseases.

Localisation	Compartment	Total	Juvenile	Middle-aged	Old
stomach	la pro	1.13	0.60	1.31	0.98
duodenum	vi	10.67	3.50	18.92	9.56
duodenum	cry-b	2.49	0.50	2.33	3.14
duodenum	jun	1.31	1.20	2.06	1.00
duodenum	cry-total	1.89	0.85	2.85	1.95
jejunum	vi	6.45	2.18	13.13	3.53
jejunum	cry-b	1.78	0.54	2.29	2.05
jejunum	jun	1.26	1.27	1.46	1.00
ileum	vi	4.61	4.00	8.54	3.53
ileum	cry-b	2.62	0.93	2.88	2.89
ileum	jun	1.96	1.36	2.50	1.96
colon	cry-a	0.80	0.25	1.10	0.80
colon	cry-b	1.95	0.66	2.00	1.98

Medians of Foxp3+ lymphocytes per group are given as cells/62 500 μm^2^. Total = all dogs disregarding their age. la pro = lamina propria. vi = villus. cry-b = basal crypt area. jun = villus-crypt junction. cry-total = total crypt area. cry-a = apical crypt area.

In healthy dogs disregarding their age, villus lamina propria Foxp3-positive cells were significantly decreased in the jejunum and the ileum compared to the duodenum (*p *< 0.001, Figure 3e). In contrast to this, differences between the localisations of the small intestine were neither detected in the basal crypt area nor the villus-crypt junction.

Furthermore, canine intestinal Tregs revealed differences in their staining intensity for Foxp3 independent of their localisation, i.e. some lymphocytes revealed weak nuclear staining and some lymphocytes had strongly labelled nuclei.

### Age-related changes in canine intestinal Foxp3 expression

In healthy dogs, the number of villus Foxp3-positive lymphocytes were significantly increased in middle-aged dogs compared to juvenile as well as old animals in the duodenum and the jejunum (*p *< 0.05, Figure 3f-h). In the basal crypt area, an increase in Foxp3-expressing lymphocytes was detected in the jejunum between middle-aged and juvenile dogs (*p *< 0.05). In the animals examined, no significant differences in Foxp3 expression between different age groups were present in the villus-crypt junction, ileum, gastric fundus, and colonic lamina propria.

### Foxp3 expression in canine gastrointestinal diseases

**Figure 4:**
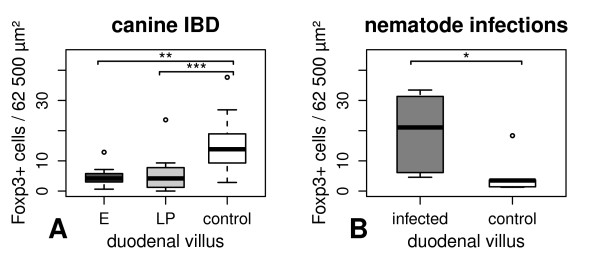
**Intestinal Foxp3 expression in dogs suffering from gastrointestinal diseases**. In canine IBD (age ranged from 2.00 to 7.22 years), Foxp3-positive cells of the duodenal villus were significantly decreased in cases with lymphoplasmacytic infiltration and in those predominated by eosinophilic inflammation compared to middle-aged controls (A). In dogs with spontaneous intestinal nematode infection (age ranged from 0.18 to 1.25 years), Foxp3 expression was significantly increased in the duodenal villus compared to juvenile healthy animals (B). E = IBD dominated by eosinophilic inflammation. LP = IBD characterised by lymphoplasmacytic infiltration. * = *p *< 0.05. ** = *p *< 0.01. *** = *p *< 0.001.

Numbers of Foxp3 positive intestinal lymphocytes of dogs with gastrointestinal diseases are summarised in Table 4 given as medians per group. In dogs suffering from IBD, Foxp3-expressing Tregs of duodenal villi were significantly decreased in cases with lymphoplasmacytic infiltration (*p *< 0.001) and in those predominated by eosinophilic inflammation (*p *< 0.01) compared to controls (Figure 4a). No differences in Foxp3 expression were detected between cases with lymphoplasmacytic and eosinophilic infiltrations. Indeed, differences in Foxp3 expression between dogs with IBD and control animals were absent in duodenal crypt area, gastric fundus, and colonic lamina propria. Regarding the Spearman's rank correlation coefficient, there was no significant correlation between the number of Foxp3-expressing lamina propria lymphocytes and the WSAVA score in the stomach, duodenum, and colon of dogs with IBD.

**Table 4:** Foxp3 expressing lymphocytes in dogs suffering from gastrointestinal diseases.

Localisation	Compartment	IBD total	IBD-E	IBD-LP	Nematode
stomach	la pro	0.85	1.00	0.70	1.90
duodenum	vi	4.13	4.37	1.85	21.06
duodenum	cry-total	1.41	1.81	0.57	3.16
colon	cry-a	1.63	2.38	1.50	2.02
colon	cry-b	2.81	3.00	1.60	1.86

Medians of Foxp3+ lymphocytes per group are given as cells/62 500 μm^2^. la pro = lamina propria. vi = villus. cry-total = total crypt area. cry-a = apical crypt area. cry-b = basal crypt area. IBD-E = IBD dominated by eosinophilic inflammation. IBD-LP = IBD characterised by lymphoplasmacytic infiltration.

In dogs with intestinal nematode infection, Foxp3 expression was significantly increased in the duodenal villus compared to controls (*p *< 0.05, Figure 4b). However, no significant differences in the number of Tregs were present in the gastric fundus, the duodenal crypt area, and the colonic mucosa in animals with parasite infections compared to healthy individuals.

## Discussion

Intestinal immune regulation including development of oral tolerance is of great importance for the maintenance of gut homeostasis [90]. Concerning this, Tregs occupy a pivotal role in cell-mediated immunosuppression [5,6]. Therefore, the aim of the study was to investigate the expression of Foxp3 in canine GALT of healthy and gastrointestinal diseased dogs.

Animals lacking clinical and pathomorphological signs of gastrointestinal diseases were used as healthy controls. Interestingly, minimal histopathological findings were present in the gastrointestinal tract of these dogs. Due to the absence of clinical and morphological correlates, these findings were interpreted as background variances of the canine gut. Although several reports on age-related changes and immunosenescence in dogs [91] would suggest an increasing inflammatory background in the canine elderly, no differences between the WSAVA scores of different age groups were observed. In contrast, WSAVA scores were significantly increased in dogs with gastrointestinal diseases compared to controls.

Immunohistochemically, application of anti-Foxp3 antibodies produced clear nuclear staining of cells revealing distinct lymphocyte morphology. Although Foxp3 has been established as a specific marker for classical Tregs further characterised by the expression of CD4 and CD25, heterogeneity of this cell population has been described [92]. In dogs, recent analyses on canine Tregs illustrate CD4-positive cells with intermediate Foxp3 expression and authors postulate that they are activated heterogeneous conventional T cells instead of Tregs [80]. In our study, differences in staining intensity for Foxp3 were noticed immunohistochemically. This suggests differences in Foxp3 expression in situ supporting the concept of canine Treg heterogeneity. However, this result could also be due to the localisation of Tregs in different tissue layers and needs to be further investigated.

Structured lymphoid tissue in the intestine plays a crucial role in mucosal immunology especially in induction and gut homing of intestinal Tregs and therefore for the establishment of oral tolerance [13,93]. The high density of Foxp3-expressing lymphocytes in those areas suggests similar roles of these anatomical compartments in dogs.

Additionally, Foxp3 was expressed by intraepithelial lymphocytes (IEL). It is well established that canine IEL are mainly characterised by the expression of CD8. However, less than 15 percent of these cells are CD4-positive [94]. Although most canine Tregs are known to express CD4, CD25, and Foxp3, a minor subpopulation of CD8-positive lymphocytes with strong Foxp3 expression is described [80]. In spite of their correct phenotype, our results confirm the immunoregulatory phenotype of canine IEL, which has recently been observed in vitro [94].

Furthermore, Foxp3-positive lymphocytes were present in the lamina propria. Concerning different compartments (vertical distribution), Tregs were significantly increased in the villus lamina propria compared to basal crypt area and villus-crypt junction. Previous studies about intestinal T cells in dogs report similar differences in their distribution [95-97]. Referring to this, a stronger exposition of the intestinal apical surface to luminal antigens is discussed. Analogously, increasing numbers of Tregs in the villus area may be related to the great importance of immunoregulation in areas characterised by high antigenic load, antigen sampling by dendritic cells, and priming of immune responses.

Additionally, the numbers of Foxp3-positive cells were significantly higher in the basal crypt area than in the villus-crypt junction. This finding was in contrast to previous observations of a constant increase in CD3-positive cells towards the villus tip [95-97]. Possibly, the increase in Tregs in the basal crypt area compared to the villus-crypt junction is due to increased numbers of IgA-secreting plasma cells towards the crypt [96] requiring a more intense local tuning of immunological tolerance in basal areas.

Interestingly, a vertical decline in Tregs was found in the colonic mucosa since the numbers of Foxp3-positive lymphocytes were significantly lower in the apical crypt area than in the basal part. Potentially, this is related to general differences of colonic Tregs in comparison to those located in the small intestine highlighting the heterogeneity of regulatory T cells. Otherwise, these differences may be due to the individual anatomy and physiology of the small and large intestines. However, further studies are needed to confirm possible differences in the vertical distribution of lymphocytes in the colonic mucosa and to evaluate its relevance.

Concerning the horizontal distribution of Tregs along the small intestinal villus, numbers of Foxp3-positive lymphocytes were significantly decreased from duodenal to jejunal as well as to ileal mucosa. In contrast to this, no horizontal differences in the expression of CD3, CD4, or CD8 have been reported [95,97]. The horizontal decline in expression of Foxp3 may be explained by decreasing amounts of food antigens progressively destroyed along the small intestine, although the numbers of bacteria progressively increase from the duodenum to the large bowel [98]. Regarding the lower frequencies of Tregs at the luminal site of the colonic mucosa compared to those in the small intestine, it is possible that food proteins are more potent generators of intestinal Tregs than commensal bacteria. In mice, it has recently been shown that T cell receptors of colonic Tregs differ from those used by Tregs in other locations [99]. Therefore, canine colonic Tregs may generally differ from those in other areas including the small intestine.

In healthy dogs, age-related changes in Foxp3 expression were observed since Tregs increased in middle-aged animals compared to juvenile as well as older individuals. Age-dependent alterations of the immune system are well-known in companion animals [91,100] and several reports on alterations of the canine gastrointestinal tract concerning different age groups exist [97,101,102]. For instance, intestinal lamina propria CD3-positive T cells and macrophages decline in older animals, whereas IgA-containing plasma cells increase with age [97]. Although age-related differences in Tregs are controversially discussed [103] and only age groups with small numbers of animals (5 to 7 dogs per age group) were examined in this study, our data suggest an increase of intestinal Tregs in middle-age dogs that may be an indication for an increase of oral tolerance in these dogs compared to juvenile individuals. Nevertheless, the numbers of Tregs may change in the canine gut during the first year of life since the canine immune system including the GALT undergoes maturation during this period. Additionally, the decline in Foxp3 expression in old animals could be a sign of immunosenescence and could possibly mean a loss of oral tolerance in the canine elderly.

Although age-related changes in Foxp3 expression were of statistical significance in this study, further investigations are needed to confirm these results using higher numbers of animals and to check the course of Tregs in juvenile dogs.

Dysregulation of mucosal immunology including a failure of oral tolerance against commensal bacteria and food proteins seems to play a crucial role in the pathogenesis of both human and canine IBD [36,104]. As it is already known, depletion of regulatory T cells leads to chronic colitis in immunodeficient mice [11]. Furthermore, intravenous application of Tregs can cure established intestinal inflammation in animal models for experimental colitis [105,106]. In Long-Evans Cinnamon rats (deficient in thymocyte development) showing spontaneous development of IBD-like colitis, Tregs are significantly reduced in the colonic lamina propria [9]. Interestingly, Foxp3-positive Tregs are present at higher densities in the lamina propria of human patients suffering from IBD than in healthy controls [107-109]. This indicates an impaired function of Tregs or a resistance of T effector cells to Treg-mediated suppression instead of a numerical aberrance in the Treg population. However, investigated dogs suffering form IBD revealed a significant reduction of Foxp3 expression in the duodenal villi in cases of both eosinophilic and lymphoplasmacytic inflammation compared to controls. Further studies using full-thickness intestinal samples are needed to check if similar differences in Tregs are also present in the jejunum and ileum in canine IBD. Interestingly, no differences in Treg numbers were present in the stomach and colon of dogs with IBD in comparison to controls. This may be an indication of different roles of small intestinal Tregs compared to those located in the stomach and the colon for canine IBD. Under physiological conditions, enrichment of Tregs in areas of inflammation is mediated by a negative feedback mechanism via IL-2 produced by T effector cells ensuring a balanced cooperation between protective immune responses and immunosuppression [110]. Therefore, our results may be a sign of a primary defect in Treg homeostasis leading to chronic inflammation due to a lack of immune regulation as a pathogenetic factor in canine IBD. Further studies on Treg function are needed to confirm this possibility.

Unlike dogs with IBD, dogs with intestinal nematode infection revealed increased numbers of Tregs in the duodenal villus compared to controls. Interestingly, no differences in Treg numbers were present in the stomach and colon of dogs with nematode infection in comparison to controls. Therefore, further studies are needed to determine whether this is related to the nematode species and its localisation in the gut or due to general differences in canine Tregs in the small intestine compared to those located in the stomach and colon. Intestinal parasites are known to trigger Th2 dominated immune responses and cause down regulation of immune responses ensuring a better survival in their hosts [42,111]. Particularly, suspension of Treg activity leads to exacerbated intestinal pathology and enhances parasite clearance [43,44]. Furthermore, helminth secretions are able to induce Foxp3 expression and enhance the regulatory function of T cells [46]. Therefore, our results suggest a parasite-related increase in intestinal immune regulation, although IL-2 mediated enrichment of Tregs may also be involved. Interestingly, parasites are discussed as potential strategies for the treatment of human IBD due to their ability to prevent intestinal pathology and their immunoregulatory properties [47,48].

This study elucidates that regulatory T cells are expressed in the canine GALT with a decline of Tregs from the villus to the crypt and age-related changes suggesting a progressive establishment of oral tolerance and immunosenescence including decreased oral tolerance in the canine elderly. A reduced number of Tregs in canine IBD suggests a defect in Treg homeostasis leading to a loss of oral tolerance. In addition, Tregs were enriched in the duodenal villi of dogs with intestinal nematode infection indicating a parasite-induced enhancement of intestinal immune regulation.

## Competing interests

The authors declare that they have no competing interests.

## Authors' contributions

JJ was involved in the study design and the collection and processing of material and performed the histopathological evaluation, immunohistochemical procedures, slide evaluation, statistical analyses. Furthermore he wrote the manuscript. US was involved in the collection and processing of material and its histopathological evaluation. Additionally, she importantly improved the manuscript. FL and IN were involved in collecting endoscopical samples and the study design and made substantial contributions to the manuscript. MHT was involved in the study design and in writing the manuscript and gave final approval of the version to be published. All authors read and approved the final manuscript.
